# A qualitative inquiry into the patient-related barriers to linkage and retention in HIV care within the community setting

**DOI:** 10.1016/j.rcsop.2022.100207

**Published:** 2022-12-05

**Authors:** Adati Tarfa, Kristen Pecanac, Olayinka O. Shiyanbola

**Affiliations:** a 2506 Rennebohm Hall, School of Pharmacy, University of Wisconsin, 777 Highland Ave., Madison, WI 53705-222, United States of America; b 4167 Signe Skott Cooper Hall, University of Wisconsin, 701 Highland Avenue, Madison, WI 53705, United States of America; c 2517 Rennebohm Hall, School of Pharmacy, University of Wisconsin, 777 Highland Ave., Madison, WI 53705-222, United States of America

**Keywords:** HIV, People with HIV (PHW), Social workers, Pharmacists, Community pharmacy, Social determinants of health, Retention in care, And linkage to care, ART, Antiretroviral Therapy, HHS, Department of Health and Human Services, HIV, Human Immunodeficiency Virus, LTC, Linkage-to-care, PCMH, Patient-centered Medical Home, SDOH, Social Determinants of Health

## Abstract

**Background:**

People with the Human Immunodeficiency Virus (PWH) experience barriers to care within the community that impedes their progress from when they discover that they are HIV positive to becoming virally suppressed. For individuals with HIV to achieve sustained viral suppression, they must be linked to care to start receiving anti-retroviral therapy and remain retained in care for continuous treatment. However, HIV surveillance data shows that many PWH are not linked to care and become lost to continuous follow-up care. Although pharmacists, PWH, and social workers interact with one another and are aware of their roles in HIV care, their perspectives on barriers to linkage and retention in care have not been investigated collectively.

**Objectives:**

Explore the perspectives of PWH, pharmacists, and social workers on barriers to linkage and retention of HIV care within the community setting.

**Methods:**

Convenience sampling was used to recruit 15 stakeholders (five PWH, five community pharmacists, and five social workers) who participated in 1-h, semi-structured interviews based on three domains of the Patient-centered Medical Home Model including (1) experiences (individual and system-level barriers to care experienced by PWH), (2) activities (social workers and pharmacists initiatives that impact adherence to care)and (3) interventions (critical issues pharmacists can address in the community to engage PWH in their HIV care). We conducted a directed content analysis based on deductive coding. To establish rigor, we focused on Lincoln and Guba's criteria of rigorous qualitative methodology: credibility, dependability, confirmability, and transferability. Similarities and divergences of themes were discussed during data analysis and agreement was reached before interpretation.

**Results:**

Emergent themes uncovered barriers to linkage and retention in HIV care as HIV-related stigma, having mental health illnesses including a history of substance abuse and social determinants of health such as homelessness, food insecurity, and insurance issues.

**Conclusion:**

The perspectives of pharmacists, social workers, and PWH can provide insight into barriers that should be identified and addressed in people living with HIV to enhance their linkage and retention in care.

## 1 Background

Despite the significant progress that has been achieved in Human Immunodeficiency Virus (HIV) management to date, the current trends in diagnoses are antagonistic to the United States Department of Health and Human Services (HHS) goal to end the HIV epidemic by 2030.^1^ The initiative aims to reduce new HIV infections in the U.S. by 90% by 2030, however, the annual number of individuals receiving an HIV diagnosis declined only by 16% during the past decade, from 44 716 in 2009 to 37 428 in 2018.^2^^,^^3^ Approximately 80% of new HIV infections are transmitted from people who are either unaware of their HIV status or are aware but are not receiving care.^4^ The path a person newly diagnosed with HIV takes to achieve optimal health outcomes is described as a Continuum of Care, which includes Diagnosis, Linkage-to-care, Retention in Care, Initiation of Antiretroviral Therapy (ART), and Viral Suppression.^5^^,^^6^

Linkage-to-care (LTC) is the first step a person newly diagnosed with HIV takes towards initiating HIV care.^7^, ^8^, ^9^, ^10^ During the linkage to care stage, healthcare professionals assess patients' needs and their barriers to care before initiating HIV medications to suppress the virus from replicating.^7^ Retention in HIV care includes clinical support to enhance patient adherence to treatment, prevention, and management of HIV-related infections, and addresses patient-specific challenges of living with HIV.^11^^,^^12^ Retention in HIV care ensures that as individuals' HIV needs are changing due to the chronic nature of the illness, their needs are met by the healthcare system.^12^^,^^13^ Despite the exigency of linkage and retention in care for people with HIV (PWH) for every 100 adults and adolescents with diagnosed HIV in 2019, only 76% were linked to care, fewer than 56% were retained in care, and only 66% of those individuals achieved viral suppression.^14^ HIV care is considered successful when an individual becomes virally suppressed and cannot transmit the virus.^4^^,^^15^^,^^16^ However, to reach viral suppression, it is paramount that individuals are supported at each stage of the HIV continuum of care. Our study focuses on the first two steps of the continuum, Linkage, and Retention in HIV care.

Studies have shown that the setting a person tests for HIV has an impact on whether or not they become linked to care, begin HIV treatment, and achieve viral suppression.^17^ In 2016, the Centers for Disease Control and Prevention promoted community pharmacy settings as feasible sites for HIV testing because of their accessibility and low potential for stigmatization compared to traditional HIV testing sites.^18^^,^^19^ Following the implementation of HIV testing in pharmacies, studies have examined the barriers to HIV testing, however, barriers to linkage and retention in care remain underexplored in those settings.

To decrease the rates of new HIV diagnosis and increase linkage and retention in care for PWH, addressing the barriers to linkage and retention in care within the community have the potential of augmenting the HHS's goal to end the HIV epidemic. Pharmacists as medication experts play a vital role along the HIV continuum of care^20^ and can critically examine the barriers to care that impact their patient's ability to become linked to and retained in care. Pharmacists have expressed willingness to help patients who may benefit from HIV-related services.^21^ Additionally, studies have highlighted the value of collaboration between pharmacists and other healthcare professionals in providing quality HIV care.^22^ Our study uniquely adds the perspectives of social workers who are trained to facilitate linkage and retention in care^23^ and often work closely with pharmacists to address patient care needs.^24^^,^^25^ The perspectives of pharmacists, social workers, and PWH are important for addressing barriers to care that impede progress towards ending the HIV epidemic.

### 1.1 Objective

The goal of this study is to explore PWH, pharmacists, and social workers' perspectives of barriers to linkage and retention in care in HIV care in the community setting.

## 2 Methods

### 2.1 Study design

PWH, pharmacists, and social workers' perceptions of barriers to linkage and retention in HIV care were explored using an exploratory descriptive qualitative approach.^26^ The study utilized a directed content analysis approach^27^ to provide knowledge of barriers to linkage and retention in care. Directed content analysis is appropriate when an existing theory or research literature can guide and identify key concepts or variables of interest.^27^^,^^28^ The Health Sciences Institutional Review Board at the University of Wisconsin–Madison approved the study procedures (2018–0657).

### 2.2 Theoretical framework

The patient-centered medical home model (PCMH) was used to guide the study design, data collection, and data analysis process. The model is usually used to design interventions that promote comprehensive care, patient-centered care, coordinated care, and accessible services for PWH in the community.^29^^,^^30^ PCMH emphasizes efficient care transitions that PWH experience along the continuum of care as they move from testing positive to achieving viral suppression. The model connects interdisciplinary teams to all aspects of a patient's well-being.^31^ In this study, the PCMH model was adapted and categorized into conceptual domains to explore barriers to HIV care from PWH, pharmacists, and social workers' perspectives. The domains of the adapted PCMH conceptual framework are experiences, activities, and interventions that occur in care for PWH and the barriers encountered within each domain.

### 2.3 Sample and recruitment

Engaging three stakeholder groups required several recruitment procedures*. PWH* were recruited to participate in the study if they self-identified as being diagnosed with HIV and were over the age of 18 years old. When recruiting potentially at-risk populations, creating partnerships with providers is an effective recruitment strategy.^32^ As such, infectious disease physicians, nurses, and pharmacists were contacted by the study team to recruit their patients with HIV.

*Community pharmacist*s are clinically trained healthcare professionals who practice in a community setting.^33^ Pharmacists were recruited through word-of-mouth and from an HIV resource center directory maintained by a medical clinic in the Midwest. The researchers actively sought out community pharmacists with experiences in taking care of PWH to provide richer descriptive data based on their professional experiences.

*Social workers* were recruited through a midwestern state Linkage-to-Care Specialist Program. The program trains social workers in HIV counseling, testing, and referral. Their training program covers tenets of case management such as motivational interviewing and expert knowledge on insurance and benefits programs.^34^

### 2.4 Data collection

The interviews were guided by the adapted PCMH model to explore the stakeholder's perspectives on barriers to linkage and retention in care for PWH. To gain a detailed response from participants each open-ended semi-structured interview guide was tailored to the group's experiences and activities related to HIV care in compliance with the domains of the PCMH model. To limit the amount of sensitive information collected from the study participants, only limited demographic information about their gender, race, and professional experiences was collected.

Semi-structured one-on-one interviews were administered face-to-face or over the phone for 60 min by a member of the study team, AT, who is a pharmacist. All study participants had the option to set up an interview time, select a private interview location such as a private room in their pharmacy for a face-to-face interview, or opt for a phone interview. Appendix A shows a sample of the interview questions for social workers mapped to the patient-centered medical home model constructs: experiences, interventions, and activities. Probing techniques were used to encourage openness and sharing of useful information with the participants.^35^

### 2.5 Data analysis

#### 2.5.1 Data transcription

The interviews were audio-recorded with an encrypted audio recorder and transcribed verbatim by a professional transcriber.

#### 2.5.2 Coding and categorization

The researchers conducted directed-content analysis^27^ of the interview transcripts using the qualitative research data management software, NVivo 10 (QSR International-Melbourne), to categorize and organize identified themes. The researchers developed a codebook using the PCMH constructs: experiences, activities, and interventions to categorize general themes that emerged from the initial coding process^37^ of reviewing the transcripts. Two researchers coded the data independently and met to discuss the codes and themes. Similarities and divergences from each transcript were discussed among the researchers. The researchers allowed for an inductive approach for the identification of new themes that did not fit into the PCMH model.^28^ The researchers expanded the themes and organized them using the aims of the study. After consensus was reached on each transcript, the results were interpreted.

### 2.6 Research rigor

Rigor was demonstrated through the establishment of the Lincoln and Guba criteria of the rigorous techniques and methods for gathering and analyzing qualitative data: credibility, dependability, confirmability, and transferability.^38^ Data saturation was achieved with 15 participants.^36^ *Credibility* refers to how trustworthy the data38 are in presenting the true experiences of participants and can be established through the researcher's prolonged engagement with the research topic and participants.^39^ Researchers AT and KP are trained as a pharmacist and nurse respectively and have participated in the caretaking of PWH in their clinical roles. AT formed a relationship with PWH in non research settings by participating and volunteering in social events within the HIV community. AT visited pharmacists and social workers in their work environments to further engage with them to gain insight into their experiences in providing HIV care. Member checking^39^^,^^40^ is another method of ensuring the credibility of a study by returning the findings to the study informants to see whether the findings accurately captured their interview responses. Member checking to validate our data analysis was performed with a subset of interview participants.^41^ Our interpretation of 1 the interview data was judged to be accurate and complete by one person with HIV, one social worker, and one pharmacist. *Dependability* is achieved once researchers have demonstrated credibility^42^ and it is established by describing the study as transparently as possible^43^ to ensure the findings are consistent and repeatable. The researchers designed a detailed description of the study procedure, which includes information such as the principles and criteria used to select participants.^43^ This information was provided to the Institutional Review Board and is available in a secured Box folder. Any changes to the procedures were documented to ensure that researchers not involved with the study can follow, audit, and critique the research process.^44^^,^^45^ *Confirmability* refers to the extent to which the study findings are participants' responses and not the researcher's biases or viewpoints.^38^ The researchers engaged in personal reflexivity^46^ through note taking during the interviews, memoing after interviews and discussing these observations during data analysis. The researchers demonstrated confirmability by providing rich quotes from the stakeholders that support the emergent themes and described how interpretations and conclusions were derived from the themes.^47^ *Transferability* refers to the degree to which the findings of the study can be applied to other contexts.^38^ The researchers thoroughly described the participants, the context of the study, and the research procedure^40^ so that the readers can determine if the results can be transferred to other settings or people.

## 3 Results

### 3.1 Participants

Fifteen participants completed the study. Five were pharmacists who practiced in community settings and their experiences from two years to thirty years of providing HIV care. One of the pharmacists completed an HIV community pharmacy residency program. Five PWH participated in the study, and two of five of the participants identified their race as African American. Only one of the participants with HIV was female and four participants identified their gender as male. All PWH that were interviewed reported using an outpatient community pharmacy for their medication management. All five social workers that participated in the study identified their gender as female. Two out of five of the social workers were bilingual with Spanish as their second language.

### 3.2 Perceived barriers to HIV care

Participants described six major barriers to HIV care: HIV-related stigma, mental health illnesses, substance abuse and misuse, insurance issues, food insecurity, and homelessness. Each of the six identified barriers to care is discussed below with a few representative verbatim quotes from the study participants.

Participants provided insight into how the themes they described impact linkage and retention in HIV care.

1. *HIV-related stigma*

People that are newly diagnosed with HIV may worry about stigma and that makes them refuse to come to terms with their diagnoses. Stigma, therefore, can inhibit a patient from taking the first step on the HIV continuum of care which is linking to care. Stigma is also connected with other barriers to care such as mental health issues and homelessness.

*“For some people, there is a great deal of stigma and shame or guilt around their HIV* diagnosis.” *[Social Worker 3]*

*“[…] if they're newly diagnosed, they may become homeless because they're living with family. And family, you know, won't take kindly to hearing this information.” [Person with HIV 3]*

2. *Homelessness*

Those who are homeless do not have a steady supply of their HIV medicines. Pharmacists and social workers described how homelessness is an issue that can prevent PWH from accessing care.

*“[…] the big ones would probably be like the homelessness, so not having permanent shelter, food insecurity. Sometimes drug use for people as well, substance use. Sometimes mental health can tie into that as well.” [Social Worker 5]*

*“If people are homeless, if people have unreliable food or food insecurity, just, if they don't have insurance if they are unemployed if they are uninsured, underinsured. Of course, stigma, fear of disclosure of status, so stigma around the disease itself can prevent people from accessing care.” [Community Pharmacist 2]*

3. *Food insecurity*

Social workers ensure their patients with HIV have access to food. It is not uncommon for PWH to have food insecurity.

*“I think with addressing all of those other things that get in their way of, so really addressing stress, really addressing like all of those other things like if we can, like food insecurity is common […]” [Social Worker 1]*

4. *Mental illnesses*

PWH may also be experiencing mental health issues. It is not uncommon for people to experience depression after testing positive for HIV. Their depressive state can hinder them from linking to receiving HIV care. A patient in this study stated their experience with depression made them stay at home and avoid people for the first two years after they found out that they had contracted HIV.

*“I stayed in this really dark depression for like two years, wouldn't come out of the house.” [Person with HIV 4]*

Pharmacists and social workers also recognized that PWH who have mental health issues may be linked to care but not adherent to their HIV care. They may not show up for medical appointments or may refuse to take the HIV medicines that are prescribed to them.

*“If someone has a cognitive delay or a chronic persistent mental illness, that may make them more at risk for HIV acquisition, and then more at risk for poor adherence to HIV medical care”. [Social Worker 4]*

5. *Substance abuse and misuse* Participants recognized that PWH who use recreational drugs are difficult to retain in care due to the unpredictable nature of addiction.

*“So a lot of it is people doing street drugs who just disappear.” [Social Worker 4]*

Besides difficulties in testing and linking to care, people that use drugs also have difficulties staying adherent to their HIV care. Care providers experience difficulties in reaching people who have a history of drug abuse for follow-up appointments.

*“I think the hardest group maybe to reach are the IV (intravenous) users.”* [*Community Pharmacist 3]*

6. *Insurance issues*

Insurances are used to mitigate the costs associated with HIV care, especially antiretroviral therapies. Insurance issues include high copays, difficulties in insurance applications, and some medication not being covered by insurance.

*“They have meds that are extremely expensive. So and it's crucial that they take those meds, and then you have insurance companies that won't pay for lost meds.” [Community Pharmacist 4]*

*“There is the cost. There are insurance companies not wanting to pay for drugs. There are pretty high copays for some people.” [Community Pharmacist 3]*

## 4 Conclusions

The PCMH guided our research to reveal barriers to linkage and retention in HIV care. PWH experience shame, stigma and guilt related to their diagnosis. Family members with HIV-related stigma have led PWH to become homeless. PWH who are homeless face additional challenges such as food insecurity. Pharmacists also revealed other insurance-related issues such as insurance companies not paying for medications and patients are left with high copays. Other barriers PWH experience include substance use to cope with their diagnosis. Mental health illness was identified as an additional risk for HIV infection and also a barrier to PWH receiving care.

The findings are consistent with Social Determinants of Health (SDOH), which are conditions that people live in that may impact their health outcomes including lack of transportation, unstable housing, and lack of health insurance that adversely impact health outcomes.^48^ The PCMH model highlights the necessity of integrating the management of SDOH to improve coordinated care.^49^ If a patient's immediate or vital needs such as housing, food, and financial needs are not met, it is difficult to ensure linkage or retention in HIV care.

In our study, participants indicated that people experiencing homelessness are at risk of not being adherent to their HIV care. Many PWH struggle with stable housing.^7^^,^^50^ In the United States, there is an average of 600,000 people that spend the night homeless, these people experience poorer health outcomes than the general population.^51^ Individuals who are homeless or in marginal housing conditions have an elevated burden of infection with HIV. Existing research suggests that HIV infections in resource-rich settings are concentrated among members of vulnerable and marginalized populations, including people that are homeless.^52^ Pharmacists have contact information on patients often linked to a primary patient address and studies have shown that pharmacists reaching people in the community leads to improved patient care outcomes for PWH.^53^ Pharmacists can work together with social workers to identify the population of PWH who are experiencing homelessness and adapt medication counseling practices that account for their housing status.

In our study, the participants linked stigma to other barriers such as mental health or homelessness. Recent research demonstrated that HIV-related stigma continues to exist in the United States.^54^^,^^55^ Stigma is defined as the undesirable or discrediting attribute a person with HIV possesses that they believe reduces their status in the eyes of society.^56^ Stigma can be perceived or experienced by a person in which they feel there are negative attitudes or discrimination towards them if their HIV status is known.^57^^,^^58^ Pharmacists can mitigate stigmatizing patient-provider interaction by training their staff on how to interact with people who are already living with highly stigmatized health conditions.

In this study, participants discussed people who use substances as being more likely to be lost to care and harder to reach for follow-up. Previous studies agree with these findings and show that people who use drugs are more likely to forget to take their medicines or are more likely to dismiss the thought of attending their HIV appointments when they are actively using drugs.^59^^,^^60^ People who use drugs are also at a higher rate of contracting HIV.^61^, ^62^, ^63^ Pharmacists can identify the patient population that uses drugs and help them to learn more about their options regarding safe use, abstinence from use, or other goals they have regarding their substance use.

Similar to substance abuse, poor mental health is both a risk factor for HIV and also associated with poor health outcomes for PWH. This study did not discuss the specific types of mental health concerns that PWH are with. However, other studies show that PWH are prone to depression, loneliness, anxiety, and psychiatric disorders.^7^^,^^56^^,^^62^^,^^64^, ^65^, ^66^ Participants in this study discussed the need for pharmacists to understand how mental health barriers inhibit people from linking to care immediately after they find out they are HIV positive or engaging in their HIV care when they are experiencing a mental health crisis. Pharmacists are encouraged to advocate for the health of PWH within their communities. Pharmacists can do this by participating in support groups that encourage PWH to take care of themselves and one another. In addition, pharmacists can participate in referral of PWH to appropriate mental health resources.

Participants discussed food insecurity as another barrier to HIV care. Food insecurity is defined as the limited or uncertain availability of nutritionally adequate, safe foods, or the inability to acquire food properly.^67^^,^^68^ Observational studies suggest that food insecurity is associated with increased HIV transmission risk behaviors and decreased access to HIV treatment.^68^ Among PWH that are receiving antiretroviral therapy, food insecurity is associated with decreased medication adherence, lack of improvement in viral suppression, and decreased survival of HIV.^67^ Clinic-to-community models to address food insecurity have used community spaces to provide food to people in need. Such community-to-patient food supply initiatives have not explored the use of community pharmacies. Only one model called a food pharmacy has been explored. The OU Food Pharmacy was developed as a pilot project between the Community Food Bank of Eastern Oklahoma and researchers at the Oklahoma University School of Community Medicine and the College of Public Health.^69^ The program was for people with chronic diseases that did not have an adequate supply of food. These individuals were provided a prescription for a monthly food supply and were provided with the food items at low discounts at a clinical location. The program had a positive impact on the blood pressure of individuals. Though such a model may not be incorporated into a community pharmacy setting, community pharmacists in our study had a food pantry as part of their pharmacy to provide food to PWH. There is a potential to examine how food pantries could be integrated into community pharmacies and to explore their impacts on patient health outcomes.

Studies have called for the integration of SDOH into the PCMH through encouraging healthcare professionals to screen patients for the SDOH, connecting patients with resources to address those SDOH, and assist patients to navigate health and social services systems.^49^^,^^70^ Social workers have expressed a desire to work closely with pharmacists to address these limiting barriers PWH experience.^24^ For pharmacists to engage in this recommendation, they first must become informed about the barriers including SDOH that their patients experience and can take strategic steps to become familiar with the resources within their community that can be used to address these patient barriers.

The strength of this study is that it qualitatively explored barriers that hinder PWH from connecting to HIV resources and continuously engaging with their HIV care from the experiential perspectives of PWH and the professional perspectives of social workers and community pharmacists. However, this study had several limitations. The study findings were based on perceptions of participants from one Midwestern US state. Barriers to linkage and retention in care might differ by regional location. Future studies will consider exploring these perceptions among other PWH, pharmacists, and social workers across different geographic locations. It is important to note that the sample size was 15 participants which is an acceptable sample size in qualitative research and data saturation was reached. However, the perspectives of other groups not well represented in the study such as women with HIV can be further explored. Future research can explore perceptions of barriers to HIV care among a more diverse group of individuals.

In conclusion, PWH, social workers and pharmacists described barriers to linkage and retention in HIV care which are considered SDOH including homelessness, food insecurity and insurance inaccessibility. Other barriers include mental health, substance abuse and misuse, as well as HIV-related stigma. Solutions for enhancing linkage and retention in care should focus on these SDOH and how they impact patient care. Future efforts to retain people in HIV care will benefit from collaboration between pharmacists and social workers tailor their services towards addressing the social needs of PWH.

## Funding resource

This research did not receive any specific grant from funding agencies in the public, commercial, or not-for-profit sectors.

## Disclosure

The authors report no conflicts of interest in this work.

## Declaration of Competing Interest

The authors declare that they have no known competing financial interests or personal relationships that could have appeared to influence the work reported in this paper.

## Appendix A Supplementary data

Supplementary material Image 1
