# Supplementary material for: A qualitative inquiry into the patient-related barriers to linkage and retention in HIV care within the community setting
Source: Explor Res Clin Soc Pharm. 2022 Dec 5;9:100207. doi: 10.1016/j.rcsop.2022.100207 (PMC9772845; doi:10.1016/j.rcsop.2022.100207)
Supplement: Supplementary file 1 — Supplementary material [file mmc1.docx]

***Appendix A: A sample of interview questions for social workers (linkage to care specialists)**

**Patient-centered Medical Home Model domain: experiences**

1. What experiences as a linkage to care specialist have you had with taking care of patients living with HIV?

2. What services do you provide to assist patients living with HIV to be linked to care?

3. What are some of the barriers you have encountered when linking patients to HIV care?

1. For barrier 1 identified, what services did you provide to help the patients?
2. For barrier 2 identified, what services did you provide to help the patients?
3. For barrier 3 identified, what services did you provide to help the patients?

4. What services do you provide to assist patients living with HIV to be retained in care?

What are some of the barriers you have encountered when offering services to retain patients in HIV care?

1. For barrier 1 identified, what services did you provide to help the patients?
2. For barrier 2 identified, what services did you provide to help the patients?
3. For barrier 3 identified, what services did you provide to help the patients?

5. What other healthcare professionals do you collaborate with to link patients to care?

1. How did you collaborate with (insert healthcare professional #1)?
2. How did you collaborate with (insert healthcare professional #2)?
3. How did you collaborate with (insert healthcare professional #3)?

6. What other healthcare professionals do you collaborate with to assist patients to be retained care?

1. How did you collaborate with (insert healthcare professional #1)?
2. How did you collaborate with (insert healthcare professional #2)?
3. How did you collaborate with (insert healthcare professional #3)?

7. What experiences have you had in working with community pharmacists to care for patients living with HIV?

**Patient-centered Medical Home Model domain: interventions**

8. How do you think community pharmacists can provide additional services to link patients with HIV to care?

1. For service #1 mentioned, how do you think community pharmacists can collaborate with you to provide this service?
2. For service #2 mentioned, how do you think community pharmacists can collaborate with you to provide this service?
3. For service #3 mentioned, how do you think community pharmacists can collaborate with you to provide this service?

9. How do you think community pharmacists can provide additional services to retain patients in HIV care?

1. For service #1 mentioned, how do you think community pharmacists can collaborate with you to provide this service?
2. For service #2 mentioned, how do you think community pharmacists can collaborate with you to provide this service?
3. For service #3 mentioned, how do you think community pharmacists can collaborate with you to provide this service?

10. What other ways do you think community pharmacists can collaborate with linkage to care specialists to care for people living with HIV?

**Patient-centered Medical Home Model domain: activities**

11. What are some of the support/services you are aware of that helps in linking patients to care?

12. What happens to a patient once they are linked successfully to care?

1. What healthcare professionals do you refer patients to for continuous support?
2. What are your thoughts on community pharmacists continuing a relationship with a patient you have successfully linked to care to make them stay in continuous care?

**Closing question**

- *Before we conclude the interview, what additional information would you like to share concerning all we have discussed?*

**If a participant wishes to discontinue the interview at any point, ask if they may be willing to share why. Thank them for their participation.**

* People with HIV and pharmacists were asked similar questions tailored to their own experiences and perspectives.
